# Light-Controlled Friction by Carboxylic Azobenzene Molecular Self-Assembly Layers

**DOI:** 10.3389/fchem.2021.707232

**Published:** 2021-08-05

**Authors:** Dandan Xue, Liran Ma, Yu Tian, Qingdao Zeng, Bin Tu, Wendi Luo, Shizhu Wen, Jianbin Luo

**Affiliations:** ^1^ State Key Laboratory of Tribology, Tsinghua University, Beijing, China; ^2^ CAS Key Laboratory of Standardization and Measurement for Nanotechnology, CAS Center for Excellence in Nanoscience, National Center for Nanoscience and Technology (NCNST), Beijing, China; ^3^ Center of Materials Science and Optoelectonics Engineering, University of Chinese Academy of Sciences, Beijing, China

**Keywords:** friction regulation, carboxylic azobenzene, self-assembly layer, nanotribology, template networks

## Abstract

Nowadays, reversible friction regulation has become the focus of scientists in terms of the flexible regulatory structure of photosensitive materials and theories since this facilitates rapid development in this field. Meanwhile, as an external stimulus, light possesses great potential and advantages in spatiotemporal control and remote triggering. In this work, we demonstrated two photo-isomerized organic molecular layers, tetra-carboxylic azobenzene (NN4A) and dicarboxylic azobenzene (NN2A), which were selected to construct template networks on the surface of the highly oriented pyrolytic graphite (HOPG) to study the friction properties, corresponding to the arrangement structure of self-assembled layers under light regulation. First of all, the morphology of the self-assembled layers were characterized by a scanning tunneling microscope (STM), then the nanotribological properties of the template networks were measured by atomic force microscope (AFM). Their friction coefficients are respectively changed by about 0.6 and 2.3 times under light control. The density functional theory (DFT) method was used to calculate the relationship between the force intensity and the friction characteristics of the self-assembled systems under light regulation. Herein, the use of external light stimulus plays a significant role in regulating the friction properties of the interface of the nanometer, hopefully serving as a fundamental basis for further light-controlling research for the future fabrication of advanced on-surface devices.

## Introduction

Reversible friction control is a topic of great interest to scientists, especially as it can help realize the control of the interface friction characteristics of microscopic surfaces. The regulation of the tribology performance from the molecular perspective has great significance for micro mechanical equipment modification to improve mechanical properties, reduce wear and prolong mechanical life (Zhang et al., 2015; Ma et al., 2016; Gao et al., 2019; Ge et al., 2019; Jin et al., 2020; Temmler et al., 2020; Zhang et al., 2020; Yang et al., 2020; Luo et al., 2021; Ren et al., 2021). In general, external field regulation is the way that people usually choose, such as light stimulation, electric field, heat, chemicals, etc. (Zhang et al., 2012; Yokoyama et al., 2014; Cai et al., 2017; Cao et al., 2018; Meng et al., 2020; Tang et al., 2020). Among all possibilities, light is probably utilized to realize intelligent control of photochromic self-assembly due to its remote control, non-invasive, and cleaning properties. As a consequence, the possibility that light stimulus can be applied remotely greatly facilitates the potential for applications where direct contact with materials is difficult or impossible (Habault et al., 2013).

The molecular ordering of photochromic compounds has attracted much attention in recent years. Photo-isomerization reaction that is able to alter the molecular properties of the building blocks without adding any chemical additives is considered to be a potential candidate for photo-responsive functional surfaces (Arai et al., 2008; Tahara et al., 2013). On top of that, organic photo-isomerized compounds can be used as the key substances to regulate the interface friction properties of a coating on the micromechanical device, with the chemical phenomena under light irradiation. Fabricating and manipulating surface polymorphism from the single molecular to the supramolecular level in determining mechanical-related properties of photo-sensitive materials has drawn persistent concern. By using the self-assembly strategy, a two-dimension self-assembled monolayer of functional molecules can be formed through non-covalent interactions (Spillmann et al., 2006). By reasonable molecular design, the ordering pattern and molecular component are able to be fine-tuned at the level of a single molecule; self-assembly of surface-confined molecules is an effective way to create a functional 2-dimensional (2D) surface. (Mas-Baleste et al., 2010; Mail et al., 2012). Self-assembly is a spontaneous organization of components into regular structures or patterns. Self-assembly has been widely used in multiple fields, such as optoelectronic materials, surface modification, nanofabrication, and life science. (Liu et al., 2021). In brief, self-assembly is capable of rendering the surface regular molecular arrangements and functional properties, which offers a useful way for the study of interfacial nano-tribology.

Azobenzene derivatives, as a prominent class of photochromic compounds, have been particularly studied due to their robustness, excellent photochromic properties, established chemistry, and light-harvesting performance (Becke et al., 1988; Jurt et al., 2006). As a test case for photo-responsive molecules, the behavior of azobenzene-functionalized systems has been extensively studied (Berson et al., 2019). Suzuki and co-workers discovered the Azo-^18^ Glue could activate ion permeation of friction-mediated dynamic disordering of phospholipid membranes by mechanical motions (Suzuki et al., 2012); The photo-responsive reversible wettability of fluorine-containing azobenzene polymers in Langmuir-Blodgett films was researched (Feng et al., 2001); Liu et al. studied the reverse switching of surface roughness in a self-organized copolymerized azobenzene monomers liquid crystal coating (Liu et al., 2015). Broer et al. established a series of Azo-LC systems liquid crystal glassy polymer coatings featuring light-driven topographical texture changes (Liu et al., 2012; Liu et al., 2013), which can potentially be utilized in controllable mechanical friction (Liu et al., 2014), wettability (Roy et al., 2016; Guselnikova et al., 2017; Huang et al., 2017), and self-oscillating/self-cleaning surfaces (Hendrikx et al., 2006). Furthermore, a “fingerprint” texture formed by the self-assembly chiral nematic polymer network coating is utilized to control the clenched friction of a robotic finger. When the “fingerprint” is turned on, the friction coefficient is decreased by a factor of four to five due to reduced surface contact. Foremost, aromatic carboxylic acids possess strong coordination capability and flexible coordination modes (Held et al., 2016; Zhan et al., 2017) since the aromatic ring in the aromatic carboxylic acid ligand has a conjugated system that is in favor of electron transfer, and the carboxylic group possesses a hydrogen-bonding effect (Li et al., 2020). Therefore, the systems bearing the aromatic carboxylic acid have the ability to form stable structures and show novel physicochemical properties. Studies on aromatic carboxylic self-assemblies have caught the attention of a lot of scientists (Aitchison et al., 2016; Das et al., 2016; Miao et al., 2016; Gao et al., 2017). The regulation of the self-assembled photo-inductive material coating by light irradiation on the surface interface, and using the properties of photo-isomerism to reversibly change the structure and properties of the coating, have broad application prospects. However, there have been few studies on the friction properties of azobenzene monolayers (Pace et al., 2007; Nalluri et al., 2011; Kumar et al., 2018).

In this work, we utilized the supramolecular templates driven by different coupling systems, which are constructed by carboxylic azobenzene self-assembly molecules with different numbers of carboxylic groups on the HOPG substrate. Additionally, we also explored the micro-friction properties of the coupling system by AFM. Herein, the template networks of azobenzene molecules were induced by hydrogen bonds and constructed on a highly oriented pyrolytic graphite (HOPG) surface. Light stimulation acts as an optical switch to regulate the structure of different adsorption layers and then to regulate the friction properties of the surface layer. The use of light external stimulus plays a significant role in regulating the friction properties of the interface of the nanometer, hopefully serving as a fundamental basis for further light-controlling research for nanotribology. We expected that the fundamental research of on-surface photo-chemistry at the level of a single molecule can considerably contribute to the future fabrication of advanced on-surface devices, simultaneously providing a pathway to fabricate certain molecular devices.

## Materials and Methods

### Materials Preparation

**SCHEME 1:**
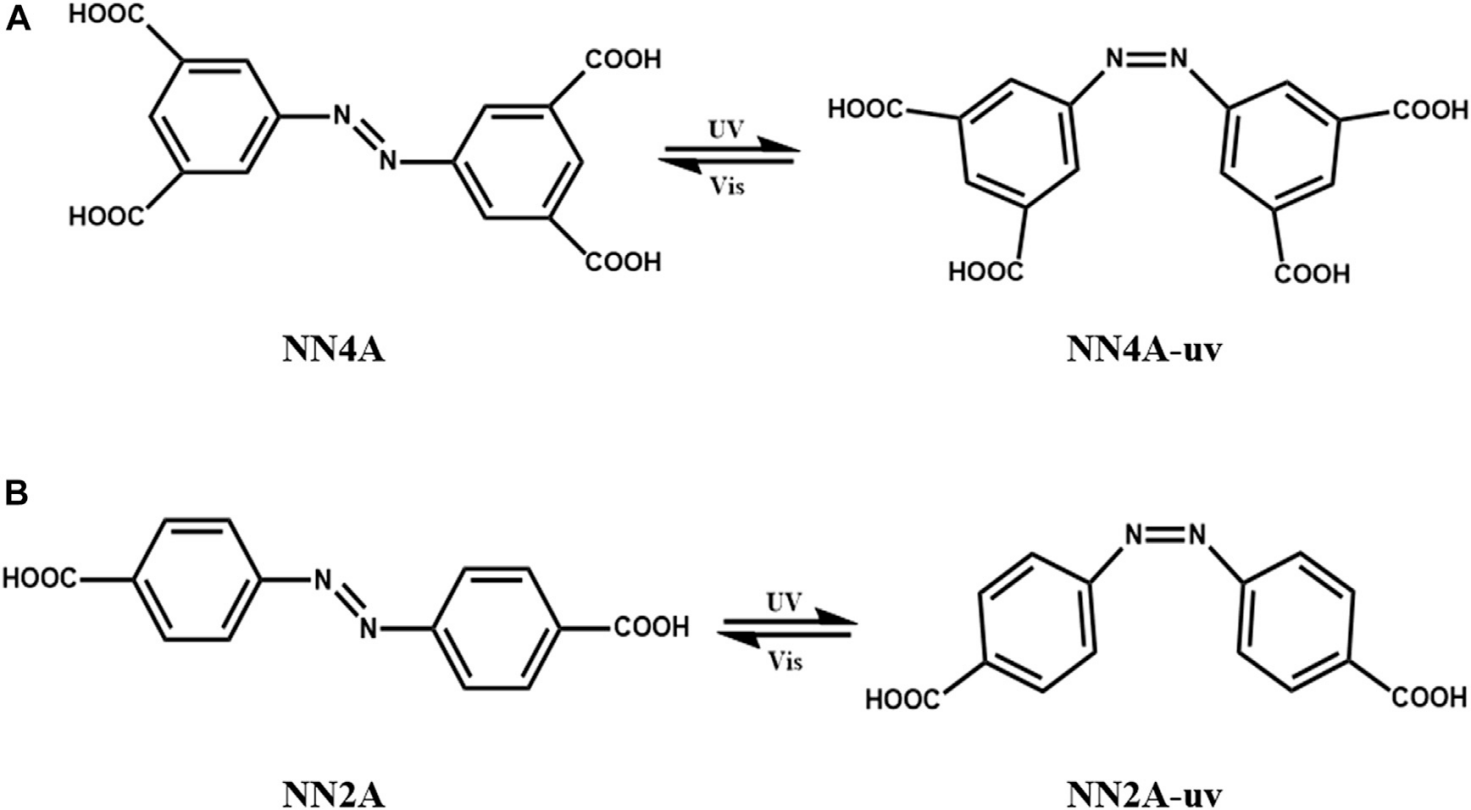
Schematic diagram of photo-isomerization of two carboxylic acids azobenzene molecules: **(A)** NN4A; **(B)** NN2A. The irradiation time of UV lamp with 360 nm is 40 min, and the power is 8 W/m.

The azobenzene derivatives, tetra-acidic azobenzene (NN4A) and di-acidic azobenzene (NN2A), as seen in Scheme 1, were synthesized by the reported process (Wang et al., 2014). Heptatonic acid was used as the solvent bought from J&K Chemical Ltd. (Beijing, China), and the solvent was used without any further purification in this work.

All the studied samples were dissolved in heptatonic acid at less than 1.0 × 10^−4^ M concentrations, then a droplet of NN4A or NN2A solution (0.5 µL) was deposited onto the freshly cleaved highly oriented pyrolytic graphite (HOPG, grade ZYB, Advanced Ceramics Inc., Cleveland, United States), forming the self-assembly samples. The samples were prepared under ambient conditions.

### Characterization and Calculation Methods

Characterization of self-assembled surface morphology was performed with a Nanoscope IIIA STM (Bruker, United States) under atmospheric conditions, and the STM images were obtained in constant current mode using a mechanically formed Pt/Ir (80/20) tip at the liquid-solid interface. The figure captions have included the specific tunneling conditions (*i.e.*, tunneling current and bias). The drift for all the images was calibrated using an atomic-resolution HOPG lattice as a reference.

In addition, we have also done a series of other characterizations of the photosensitivity of the self-assembled molecular layer and the carboxylic azobenzene, using instruments including the X-ray photoelectron spectroscopy/ATR-FTIR/Contact angle goniometry/spectroscopic ellipsometry/light scattering measurements. Supplementary Material for detailed results.

The theoretical calculation was performed using density functional theory (DFT) provided by the DMol3 code (Delley et al., 2000). Herein, we used the periodic boundary conditions (PBC) to describe the 2D periodic structure on the graphite. A self-consistent field procedure was performed with a convergence criterion of 10^–5^ on the energy and electron density. Combined with the experimental data, we have optimized the unit cell parameters and the geometry of the adsorbates in the unit cell. To evaluate the interaction between the adsorbates and HOPG, we designed the model system. In our work, we performed our calculations on infinite graphene monolayers using PBC. In the super lattice, graphene layers were separated by 40 Å in the normal direction. As for the adsorbates on graphene, we used graphene supercells and sampled the Brillouin zone by using a 1 × 1 × 1 k-point mesh (Shi et al., 2018).

The microscopic lateral forces (Friction) of self-assembly samples were measured by the MFP-3D AFM (Asylum Research, United States) with the silicon CSG10 probe (nominal normal spring constant 0.1 N/m) at room temperature. The measurement was performed at the gas-solid sample interface after the solvent evaporated. Before each measurement, the genuine normal and lateral factors need to be calibrated. The sensitivity of the normal photodetector was calculated from the slope of the force curve acquired on a stiff substrate. The power spectral density of thermal noise fluctuations under environmental conditions can be used to estimate the normal spring constant. The voltage result was converted into a force value, and the lateral factor was calculated based on the improved wedge calibration method by scanning the commercial TGF11 silicon grating (MikroMasch). In the contact mode, the microscopic friction was measured by Scanning perpendicular to the direction of the cantilever beam. The scanning area was 100 nm × 100 nm, and the scanning frequency was 1 Hz. In addition, the adhesion force was recorded the retrace signals with 15 nN load.

The photo-irradiation experiments were carried out in order to study the isomerization reversion, with UV light at 365 nm for 40 min, and the re-examined samples were illuminated by visible light at 560 nm for 20 min using the Xenon lamp (PLS-SXE 300, 50 W); the 365 nm glass filter and 560 nm filter were bought form Peking Perfect Company (CHN).

## Results and Discussion

The two carboxylic azobenzene compounds are with different numbers of carboxylic groups symmetrically locating at the end of the azobenzene molecule. Their microstructural characteristics and friction properties have been found to exhibit different photosensitive properties under light irradiation. A schematic diagram of the isomerization of two self-assembled layer material molecules is exhibited in Scheme 1.

### Photo-Isomerization of Tetra-Acidic Azobenzene and Di-Acidic Azobenzene Self-Assembly Layers

**FIGURE 1:**
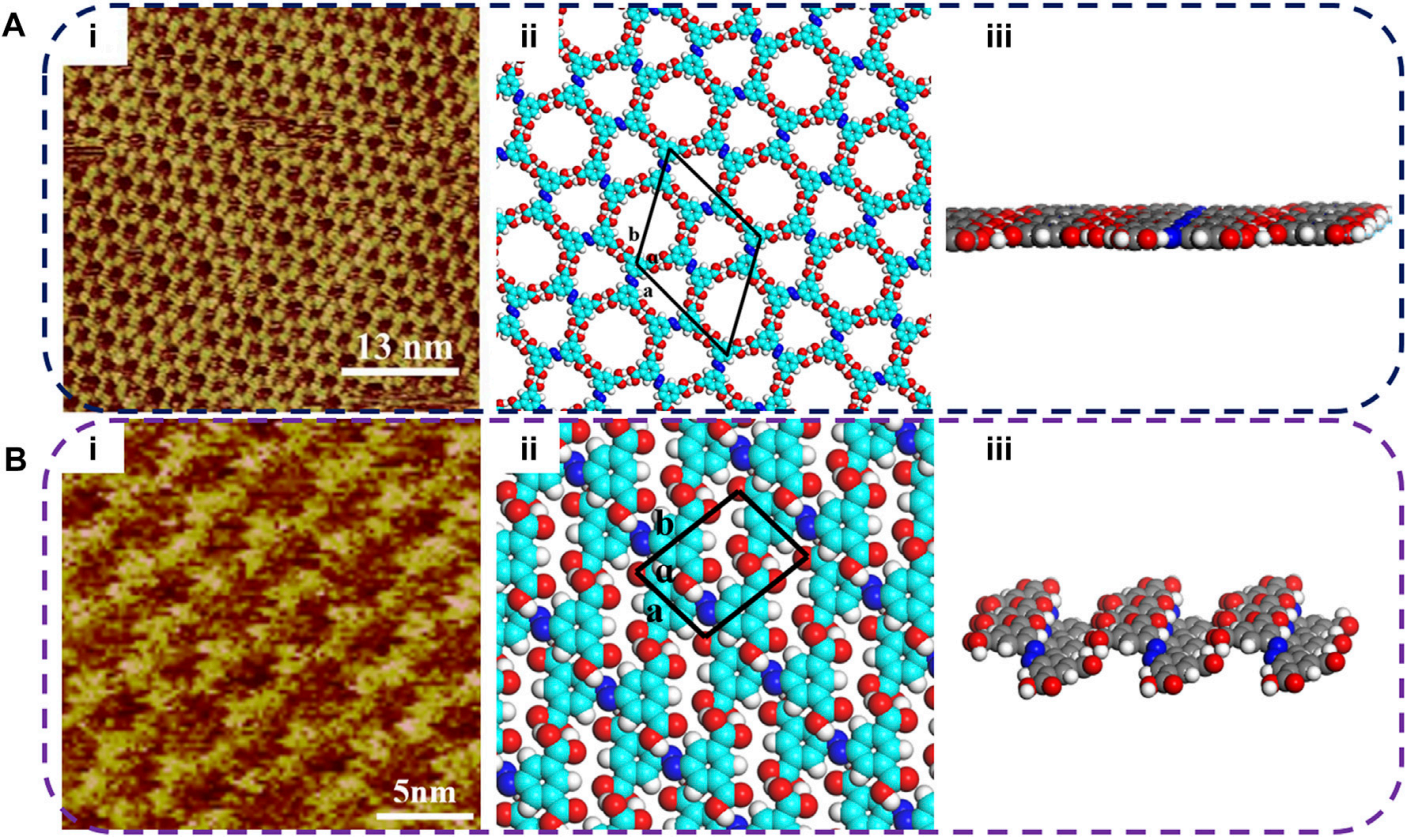
STM images of the NN4A self-assembly template networks structures at the HOPG interface under light irradiation. **(A)** NN4A: **(i)** High resolution; **(ii)** The simulated molecular packing structure; **(iii)** 3D structural front view of NN4A molecular layer. **(B)** NN4A-uv: **(i)** High resolution; **(ii)** The simulated molecular packing structure; **(iii)** 3D structural front view of NN4A-uv molecular layer. Tunneling conditions: *I*_set_ = 389 pA, *V*_bias_ = 576 mV. The irradiation time of UV lamp with 360 nm is 40 min, and the power is 8 W/m.

The self-assembled nanostructure of NN4A is shown in Figure 1. The self-assembled molecular layers of NN4A substances have been characterized in previous studies (Li et al., 2008), as seen in Figure 1A. Combined with previous research results, the assembly structure of NN4A on the HOPG surface forms a Kagome´ image in which two adjacent carboxylic groups form a pair of hydrogen bonds and make up the network structure, which included two cavities formed by six symmetry benzene rings and three NN4A molecules, respectively. As one typical photosensitive substance, NN4A will isomerize under light irradiation. The NN4A self-assembly layer on HOPG could isomerize from *trans* to *cis* isomers upon irradiation with UV light at 365 nm and reversibly isomerize from *cis* to *trans* when irradiation with visible light at 560 nm. As revealed in Figure 1B, after UV light irradiation, the Kagome networks of NN4A are broken, and liner characteristics appear in the assembled structures of NN4A-uv. Unlike the case of the NN4A, one NN4A-uv molecule is linked by two other NN4A-uv molecules, and the two adjacent NN4A-uv molecules arrange in an opposite direction via H-bonds, thus every isomerized NN4A molecule forms two pairs of hydrogen bonds, as shown in Figure 1B, the corresponding molecular model calculated by DFT method based on STM observations. The measured unit cell is also superimposed on the molecular model with *a* = 0.8 ± 0.1 nm, *b* = 1.1 ± 0.1 nm, *α* = 87 ± 1°, as shown in Table 1. In addition, it can be seen in Figures 1Aiii,Biii that the 3D structure of the molecular layer changed from the tile type to the bulge shape at the hydrogen bond connection position after being irradiated by light.

**TABLE 1:** Experimental (Expt.) and calculated (Cal.) cell parameters of NN4A and NN2A before and after light irradiation on the HOPG surface. The measurement error for the length is 0.1 nm, and that of the angle is 1°.

System	a (nm)	b (nm)	α(°)
NN4A	2.50	2.50	120°
NN4A-uv	0.80	1.10	87°
NN2A	0.76	1.48	33°
NN2A-uv	0.56	1.17	65°

**FIGURE 2:**
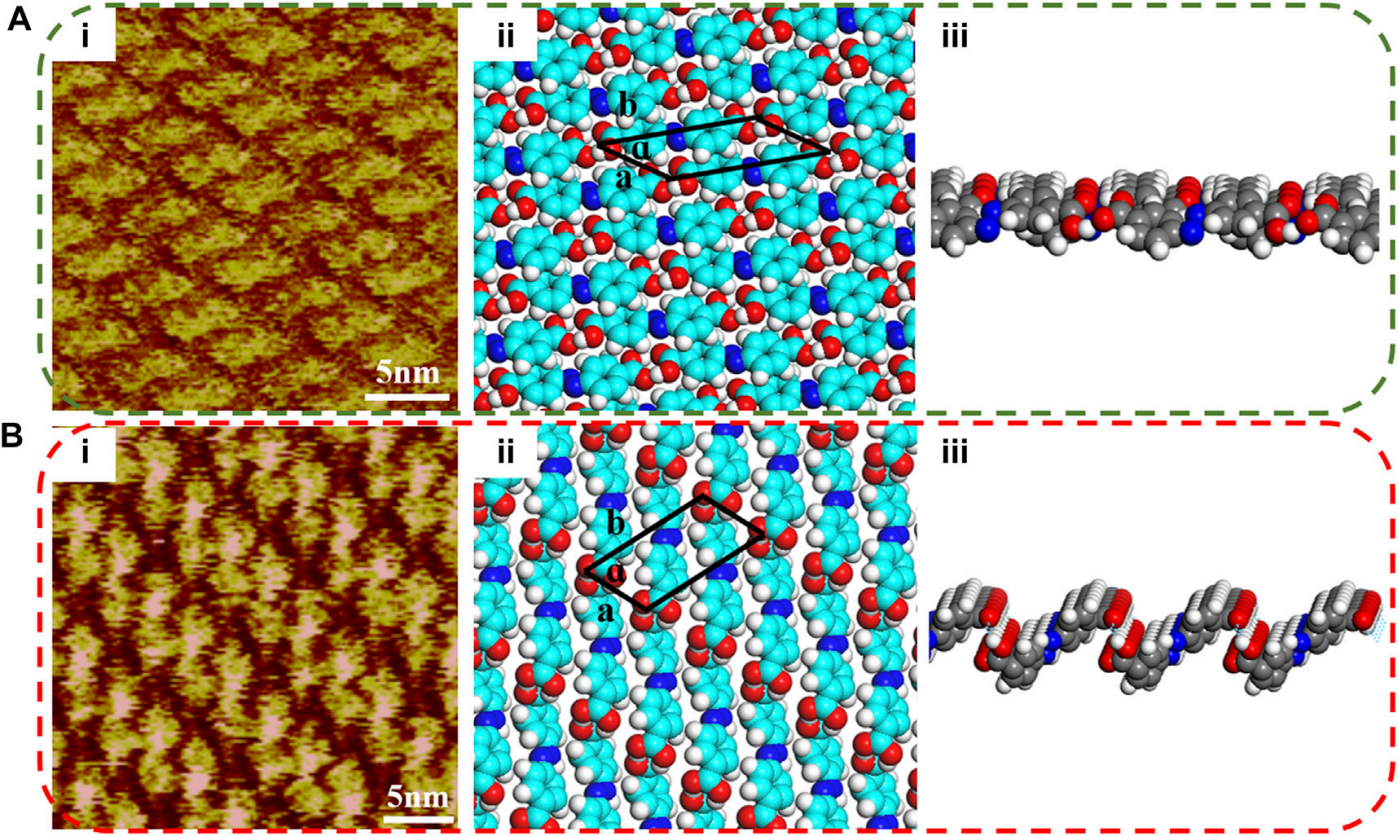
STM images of the NN2A self-assembly template networks structures at the HOPG interface under light irradiation. **(A)** NN2A: **(i)** High resolution; **(ii)** The simulated molecular packing structure; **(iii)** 3D structural front view of NN2A molecular layer. **(B)** NN2A-uv: **(i)** High resolution; **(ii)** The simulated molecular packing structure; **(iii)** 3D structural front view of NN2A-uv molecular layer. Tunneling conditions: *I*_set_ = 290 pA, *V*_bias_ = 620 mV. The irradiation time of UV lamp with 360 nm is 40 min, and the power is 8 W/m.

Another sample substance NN2A was studied. However, unlike NN4A, NN2A has only a symmetric pair of carboxylic acids groups at both ends. As presented in Figure 2A, the self-assembled molecular layer of NN2A is detected and shows an order structure: every single molecule in the region is lined up. The length and width of the unit cell are measured according to the length size of the central NN2A backbone. However, the width of the bright spot stripe of NN2A can be known to be less than that of the NN2A molecule, so every molecule is sloping on the HOPG. The unit cell parameters are calculated with *a* = 0.76 ± 0.1 nm, *b* = 1.48 ± 0.1 nm, *α* = 33 ± 1°, as shown in Table 1. In Figure 2A, the proposed molecular model of the STM image can be seen. After UV light irradiation at 365 nm, a different well-ordered network can be obtained, as shown in Figure 2B. Due to structural changes in the STM molecule image patterns, it shows good agreement with the isomer transformation from *trans* to *cis*. The distance between two molecules in the length direction becomes smaller after UV light illumination. The parameters of a unit cell in the Figure 2B model are also presented: *a* = 0.56 ± 0.1 nm, *b* = 1.17 ± 0.1 nm, *α* = 65 ± 1° (as seen in Table 1). Similarly, Figures 2Aiii,Biii show that the 3D structures of the molecular layer of NN2A also have obvious changes. The longitudinal inclined benzene ring formed through the tightly bound of the hydrogen bond between the molecules of NN2A, while the head-to-head linkage between the molecules of NN2A-uv result in the projection of the carbon and hydrogen on the benzene ring into a labile wrinkle-like structure.

All the well-ordered formed patterns are stable by virtue of the van der Waals interactions and hydrogen bonding (Zhu et al., 2019). The van der Waals interactions mentioned here mainly come from the interactions between the molecule and HOPG. Meanwhile, the hydrogen bonding is expressed as O-H...O, which is formed from an O atom in the side chain in the carboxyl group and an H atom in the carboxyl group of the adjacent molecule. The hydrogen bonding and van der Waals interactions together control the self-assembled structures, when the UV light triggers the self-assembled molecules.

### Theoretical Calculation of Two Self-Assembly Systems

Generally, the self-assembled molecule structures result from the balance of a variety of interactions, which include the molecule-molecule and molecule-substrate interactions (Zhu et al., 2019). In order to further understand the self-assembled mechanisms based on observed phenomena, we performed DFT calculations to investigate the arrangement of self-assembled nanostructures based on the related interactions. The DFT results exhibit a difference in the energies before and after light irradiation (as listed in Table 2). The total energies of NN4A self-assembled structure (−101.506 kcal/mol) are higher than those of the NN4A-uv structure (−71.704 kcal/mol), which is possibly caused by isomerization. Simultaneously, the total energies of the NN2A self-assembled structure (−41.839 kcal/mol) are smaller than those of the NN2A-uv structure (−44.597 kcal/mol). For comparison, the energies of the NN4A molecules are higher than those of NN2A, which is attributed to the stronger interactions between the NN4A molecules. Other than the intermolecular interactions, the interactions between molecules play an important role in the surface supramolecular assembly process. As shown in the first column in Table 2, the interaction energies between molecules of NN2A (−14.873 kcal/mol) are lower than those of all the others. It can be concluded that the van der Waals interaction between molecules and HOPG is strongest due to the much tighter alignment of molecules, which can evaluate the interaction strength and thermodynamic stability of the assembly systems with different unit cells.

**TABLE 2:** Energies of self-assembled systems on the HOPG surface.^a^

	Interactions between adsorbates (kcal/mol)	Interactions between adsorbates and substrate (kcal/mol)	Total energy (kcal/mol)	Total energy per unit area (kcal/mol Å^−2^)
NN4A	−58.483	−43.023	−101.506	−0.541
NN4A-uv	−33.218	−38.486	−71.704	−0.816
NN2A	−14.873	−26.966	−41.839	−0.683
NN2A-uv	−24.616	−19.981	−44.597	−0.751

a The total energy includes the interaction energies between adsorbates and the interaction energies between molecules and substrate.

### Tribological Properties of Two Self-Assembly Systems

**FIGURE 3:**
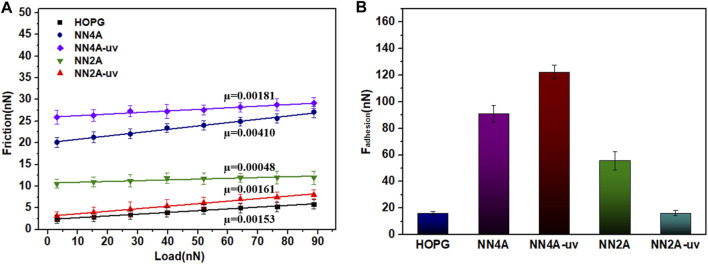
The relationship curves of **(A)** Lateral force (Friction)-load; **(B)** F_adhesion_ measured by AFM. The irradiation time of UV lamp with 360 nm is 40 min, and the power is 8 W/m.

There have been some studies on the tribological properties of the self-assembled organic molecule layers (Nalluri et al., 2011; Shi et al., 2018), while the studies on the tribological properties of the photo-regulated molecular layers have been reported rarely (Pace et al., 2007; Tang et al., 2020). For the self-assembly layers of the NN4A and NN2A molecules, we had investigated the tribological lateral forces using AFM at room temperature. Under the light stimulation, the contrast between the illuminated molecular layers and the original molecular layers produced a significant friction contrast. The experimental results of AFM friction performance are summarized in Figure 3, the lateral forces (Friction) of the NN4A-uv/NN4A/NN2A/NN2A-uv/HOPG are listed as a function of the applied load. It can be seen in Figure 3A that the lateral force of the system has a good linear fitting relationship with the load. By contrast, the lateral force of NN4A is generally higher than that of NN2A, which corresponds to the total energy in Table 1. Meanwhile, we defined the slopes of the linear fitting curves of the lateral force-load as the corresponding friction coefficient, labeling this in Figure 3A. For NN4A, the friction coefficient of the NN4A-uv structure self-assembled layer after light illumination is smaller than that of the original material NN4A, with about a 56% reduction. However, in comparison, the friction coefficient of the NN2A-uv structure after light illumination has the opposite trend, which is higher than that of the original material NN2A, with approximately more than twice. Therefore, light stimulation has a better regulation effect on the self-assembly system of NN2A. Especially, comparing the three other self-assembled systems, the NN2A has the lowest friction coefficient below 0.001, indicating it has excellent microscale superlubricity properties.

**FIGURE 4:**
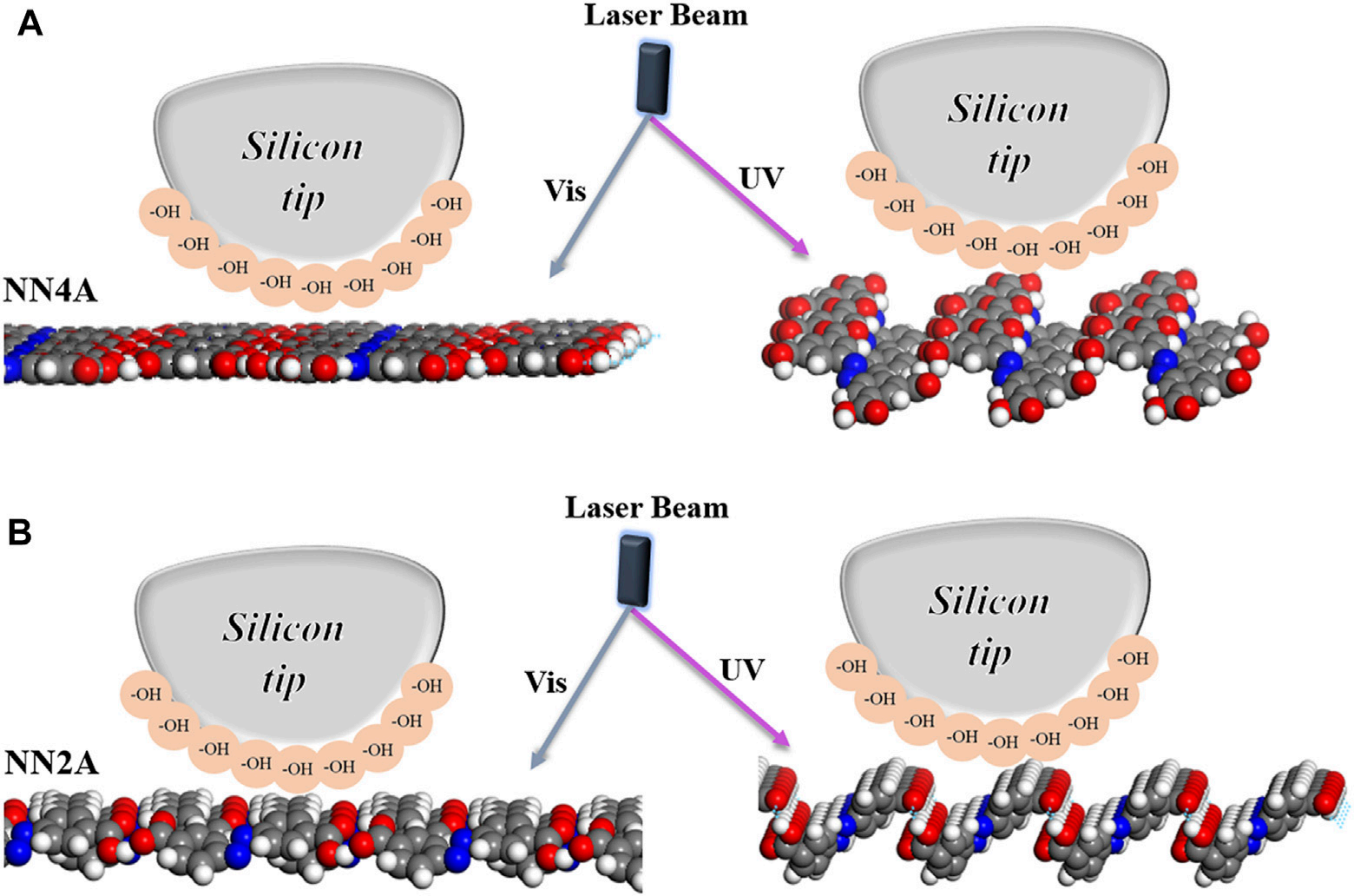
3D schematic diagram of two molecular layers configuration arrangement under AFM probe scanning. The irradiation time of UV lamp with 360 nm is 40 min and the power is 8 W/m.

Moreover, we also investigated the adsorption energy by the AFM force measurement mode to further research tribological properties of the self-assembled system (as seen in Figure 3B). As for NN4A, combining with the results of simulation analysis, the structure of NN4A-uv after exposure to light has smaller total energy values (consisting of interactions between absorbates as well between absorbates and substrate). It is mentioned here that the unit cell area of the NN4A-uv substance after light illumination is larger than that of the original substance NN4A, the obtained per unit area energy is smaller. However, by observing the 3D molecular layer structure before and after light illumination (shown in Figure 4A), it can be found that due to the increase of the fluctuation of molecular layer structure after illumination, leading to the adhesion force (F_adhesion_) between the probe and the molecular layer increases. As a result, the NN4A-uv material molecular layer after illumination has a larger initial lateral force as shown in Figure 3A.

Similar to NN2A self-assembled systems, the 3D structure of the NN2A self-assembled molecular layer transformed from the original approximately tile-like state to the state of molecular erection closely arranged after light illumination, and the energy between adsorbates increases. Therefore, more energy barriers need to be overcome when the self-assembled molecular layer comes into contact with the AFM probe, which is reflected in the increase of the friction coefficient of the system. The more important is that the hydrocarbon structures on the benzene ring in the NN2A molecular layer are exposed after light illumination (as 3D structures shown in Figure 4B), which will interact with the hydroxyl on the silicon tip to increase the lateral friction force. However, the conjugation between the benzene ring and the HOPG decreases due to the molecule isomerization, so that the adsorption energy between the adsorbates and the HOPG decreases, and the adhesion force thus decreases after light irradiation.

**FIGURE 5:**
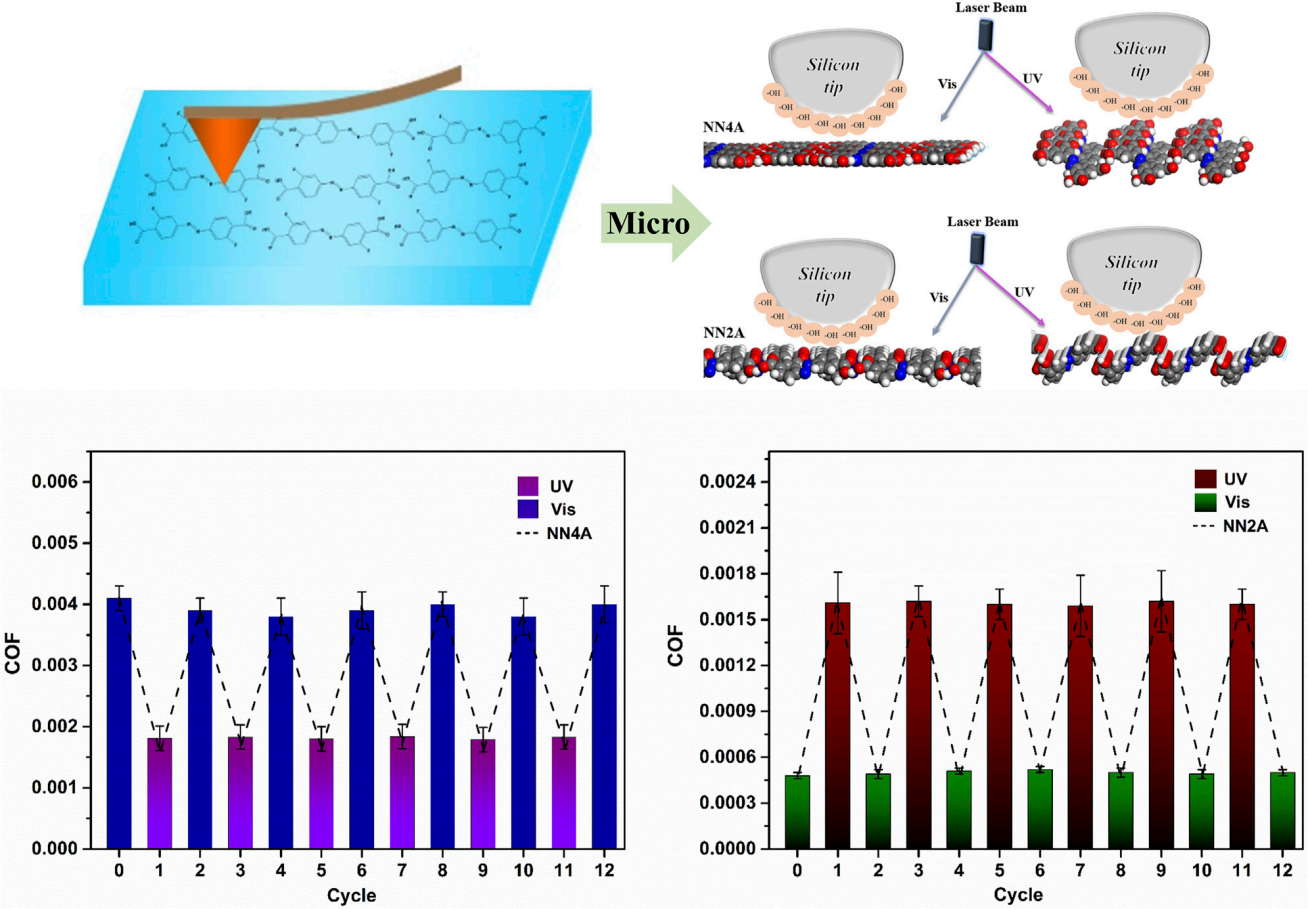
The comprehensive graphic content.

In conclusion, the experimental results indicate that light-stimulation has a good effect on the regulation of photo-isomerizable molecular self-assembly systems, and the regulation of dicarboxylic azobenzene (NN2A) has a more obvious result than that of tetra-carboxylic azobenzene (NN4A) molecule. As shown in Figure 5, the friction properties of the two self-assembled molecular layers under photo regulation have displayed excellent fatigue resistance. This has great implications for our choice to optimize the friction performance of self-assembly coatings on micro-surface interfaces.

## Conclusion

In summary, the two photo-regulated carboxylic azobenzene molecules have been synthesized and explored. We discovered that these two self-assembled molecular layers of carboxylic azobenzene photosensitive samples have excellent reversible friction properties by light regulation. In order to study the effect of light stimulation on the frictional properties, we first characterized the structure of the self-assembled molecular layer before and after light illumination by STM and calculated the adsorption energy in the self-assembled system by DFT simulation model. Then, AFM was used to explore the lateral friction force and adhesion force to observe the results of the optical control friction performance. Via light regulation, the friction coefficients of the two photosensitive samples of NN4A and NN2A are respectively changed about 0.6 and 2.3 times. By comparison, light illumination has a better regulation effect on the self-assembly system of NN2A. Especially comparing the other self-assembled systems, the NN2A self-assembled system has the lowest friction coefficient below 0.001, indicating it has excellent microscale superlubricity. Our research demonstrated that photosensitive materials can well regulate the structure of the micro surface interface and can thus regulate and optimize the friction properties. This work is beneficial to provide a theoretical basis for the exploration of light regulation in nanotribology.

## Data Availability

The original contributions presented in the study are included in the article/Supplementary Material, further inquiries can be directed to the corresponding authors.
