# Supplementary figures and images for: Light-Controlled Friction by Carboxylic Azobenzene Molecular Self-Assembly Layers
Source: Front Chem. 2021 Aug 5;9:707232. doi: 10.3389/fchem.2021.707232 (PMC8374315; doi:10.3389/fchem.2021.707232)

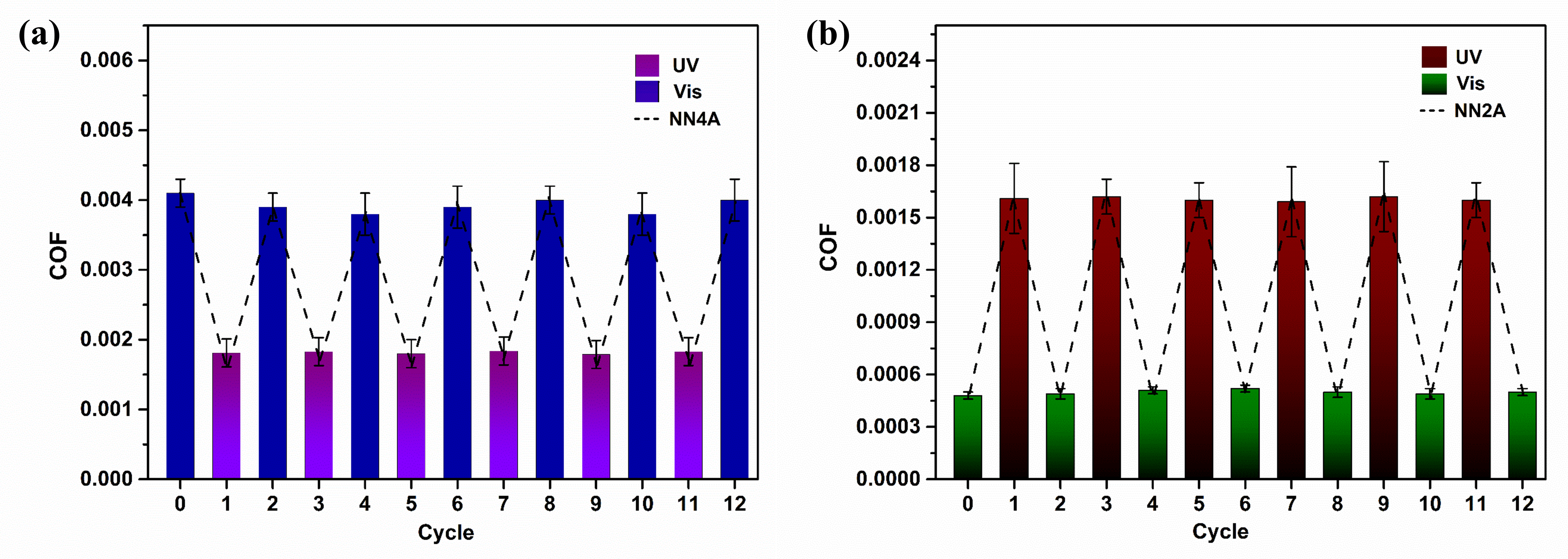

Supplement: Supplementary file 1 [file Image6.TIF]

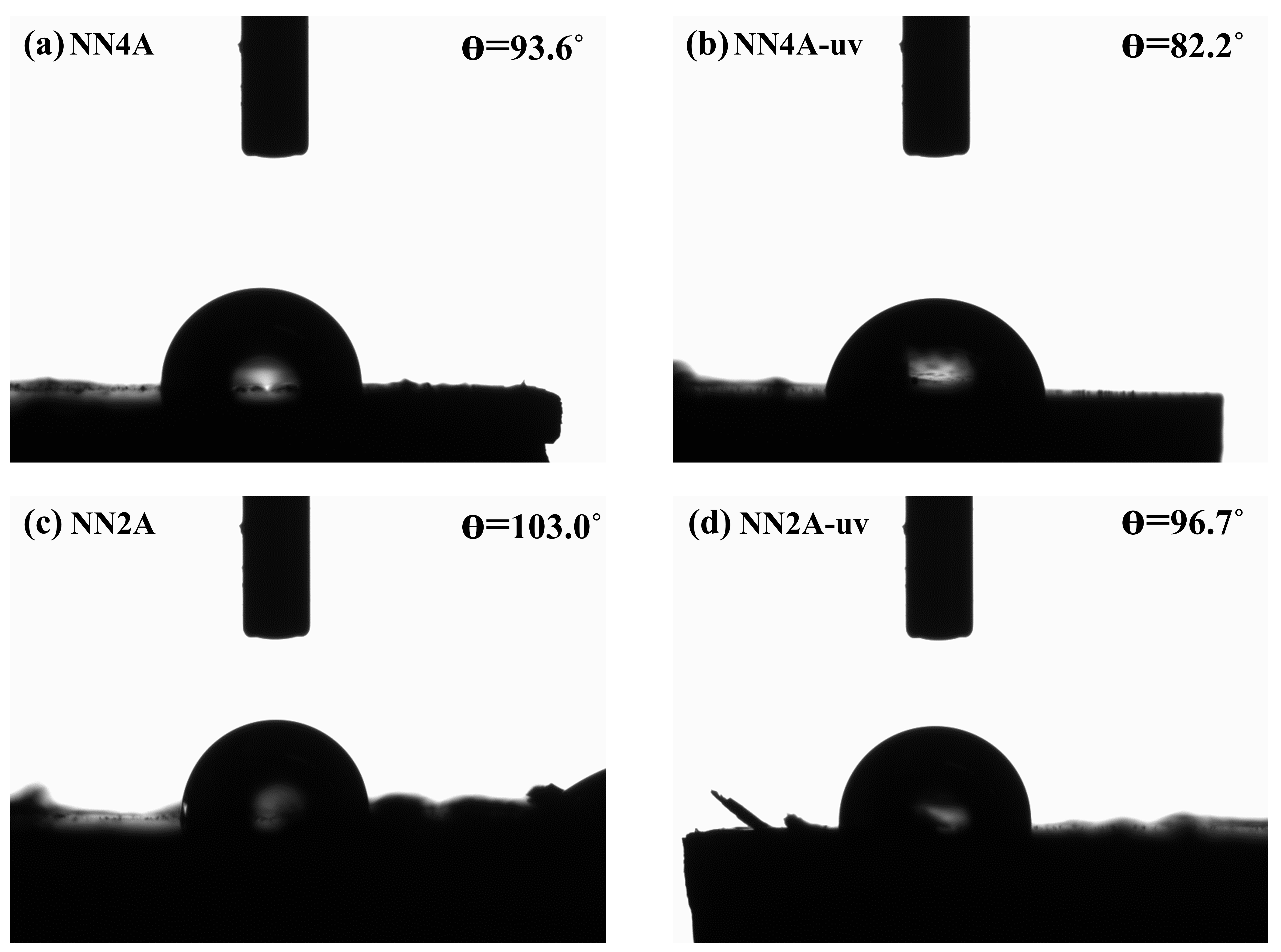

Supplement: Supplementary file 2 [file Image3.TIF]

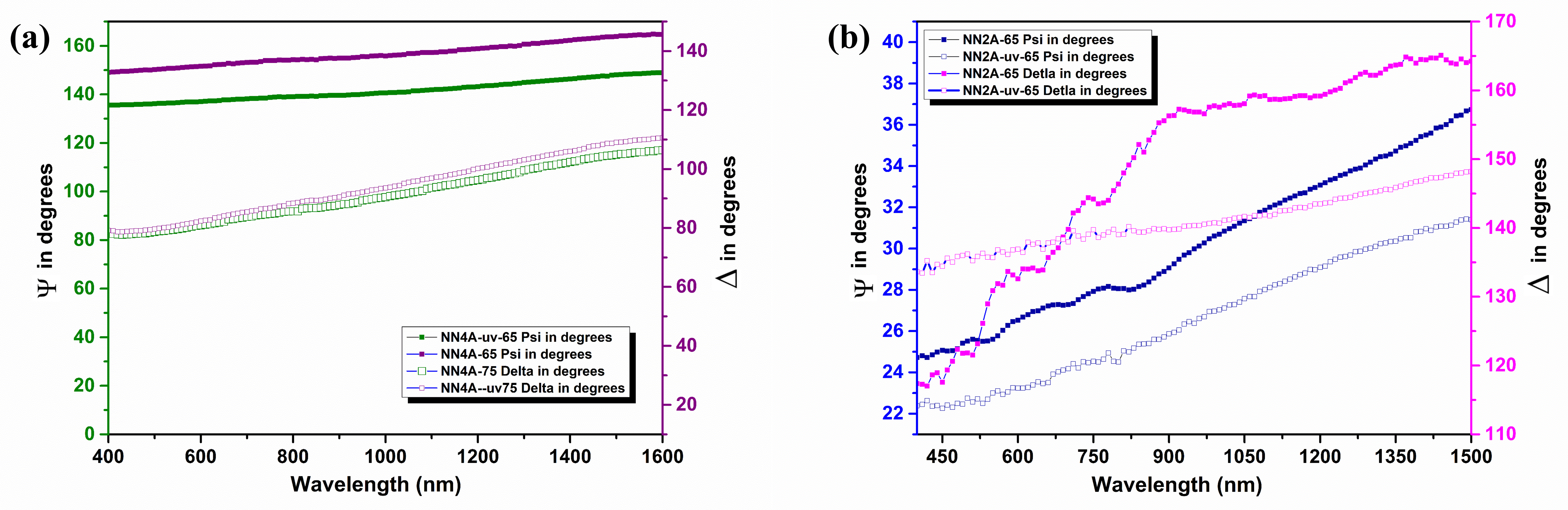

Supplement: Supplementary file 3 [file Image4.TIF]

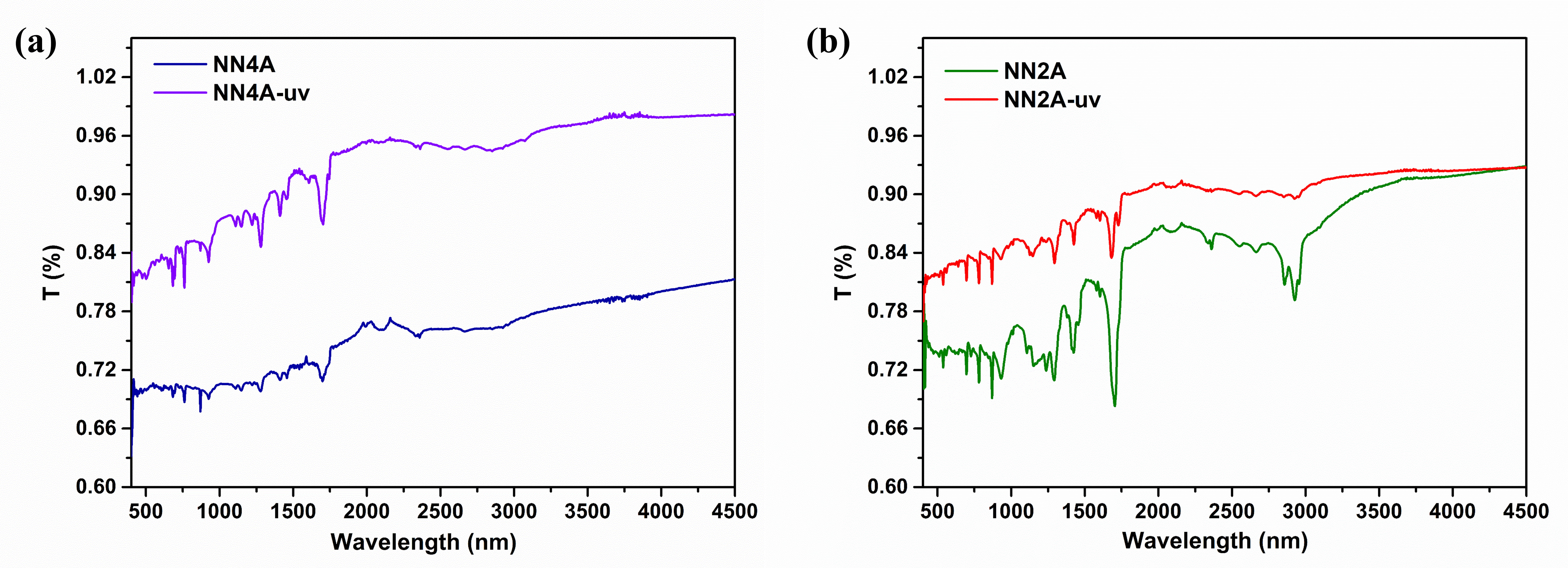

Supplement: Supplementary file 4 [file Image2.TIF]

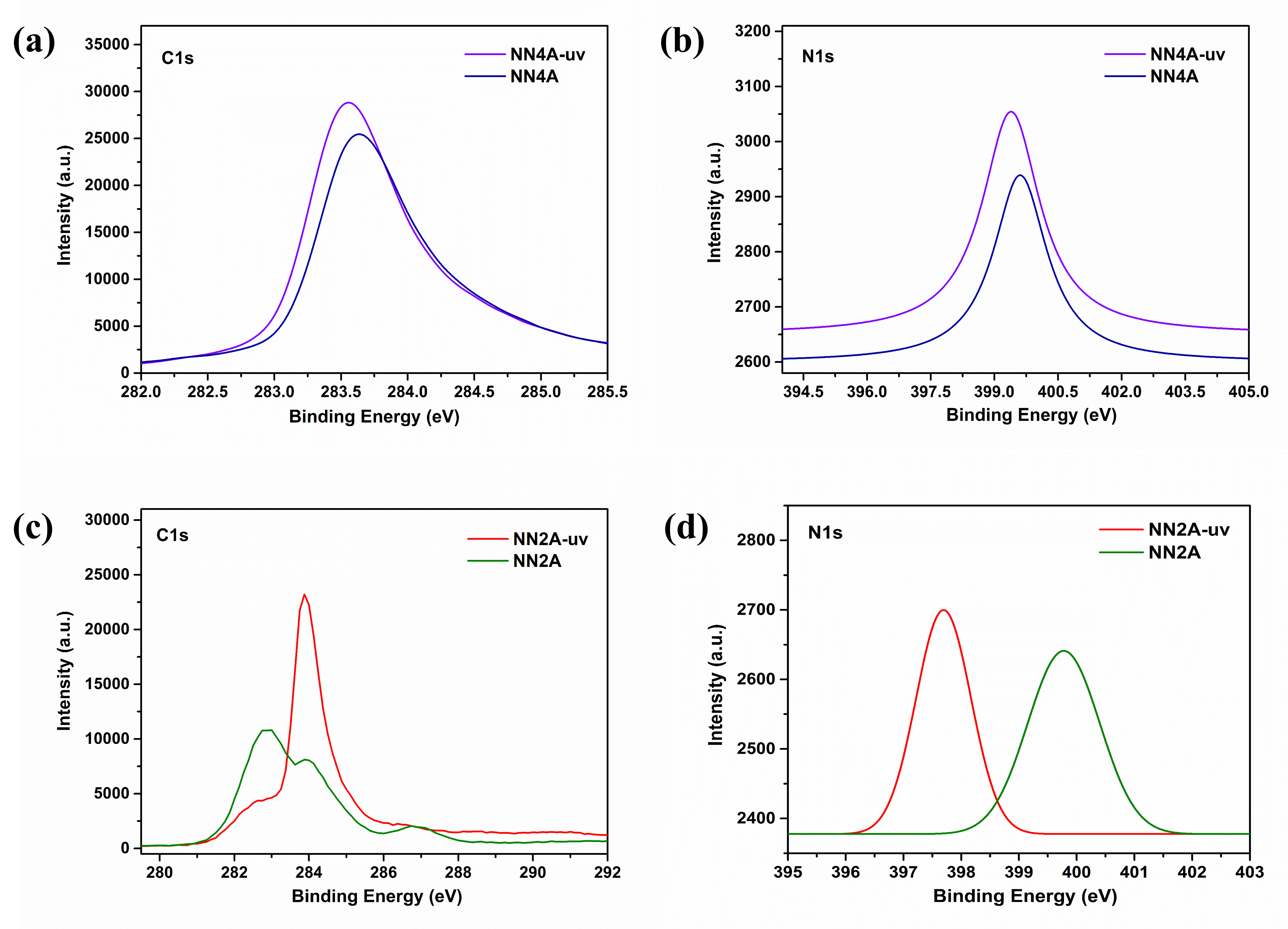

Supplement: Supplementary file 5 [file Image1.TIF]

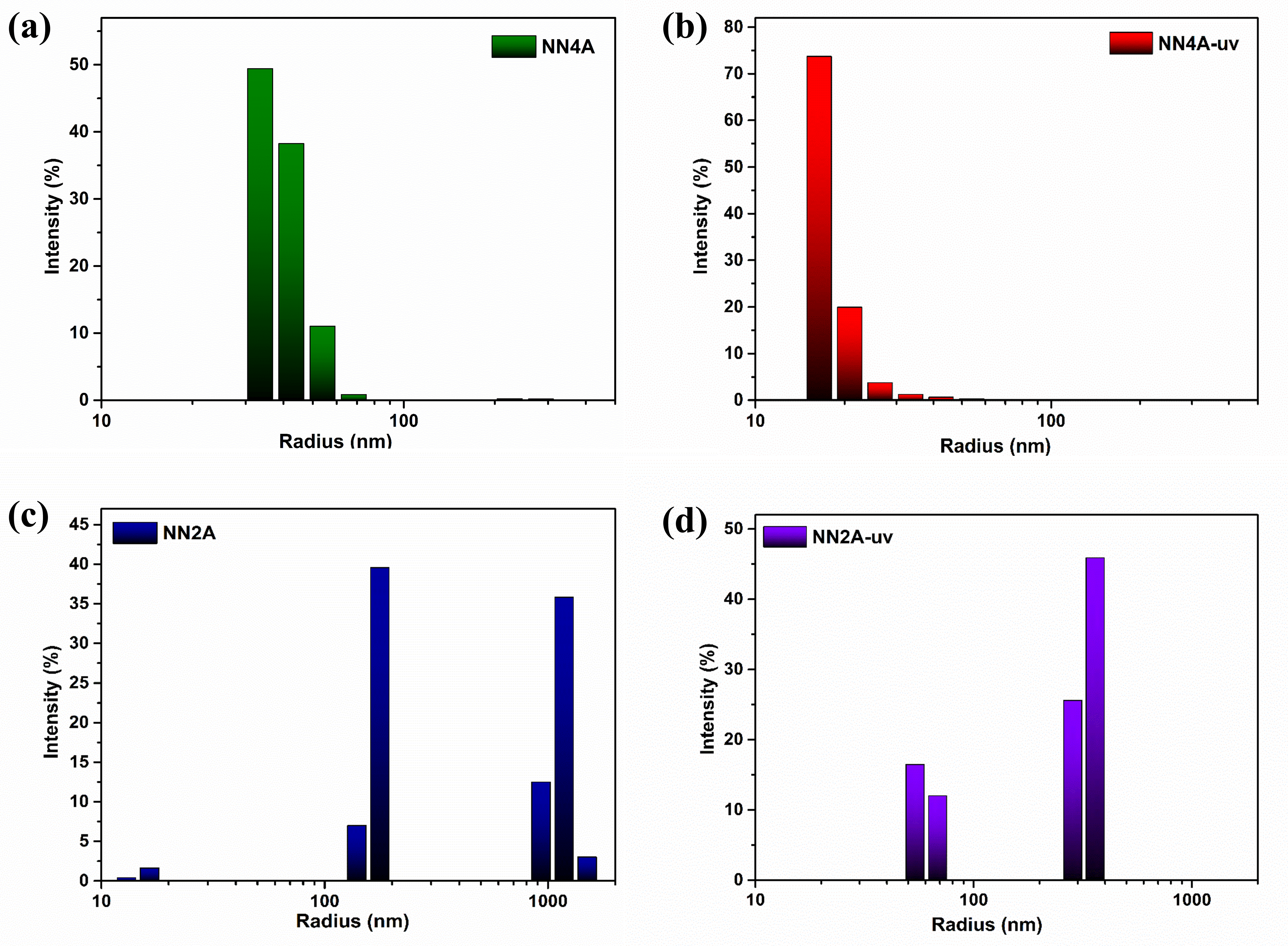

Supplement: Supplementary file 7 [file Image5.TIF]
