# Supplementary material for: Light-Controlled Friction by Carboxylic Azobenzene Molecular Self-Assembly Layers
Source: Front Chem. 2021 Aug 5;9:707232. doi: 10.3389/fchem.2021.707232 (PMC8374315; doi:10.3389/fchem.2021.707232)
Supplement: Supplementary file 6 [file DataSheet1.PDF]

Supplementary Material

1 Characterization of photo-regulated self-assembled monolayers

In addition to using STM as mentioned in the text to characterize the changes of the self-assembled surface under light regulation, a series of other characterizations of the self-assembled molecular layers before and after light regulation have been carried out to prove the changes of the molecular layers under ambient conditions.

Firstly, the photo-control of NN4A and NN2A self-assembled molecular layers were characterized by the X-ray photoelectron spectroscopy, and the results are shown in **Figure S1**. By analyzing the narrow spectrum of the C and N elements on the molecular membrane, we found that the peak values of the C and N spectrums on the NN4A membrane were shifted to the left and increased to a certain extent after illumination, while the C spectrum of the molecular membrane of NN2A showed a peak increase at 284eV and a peak decrease at 282.7eV after illumination, and its N spectrum showed a complete peak shift to the left after illumination.

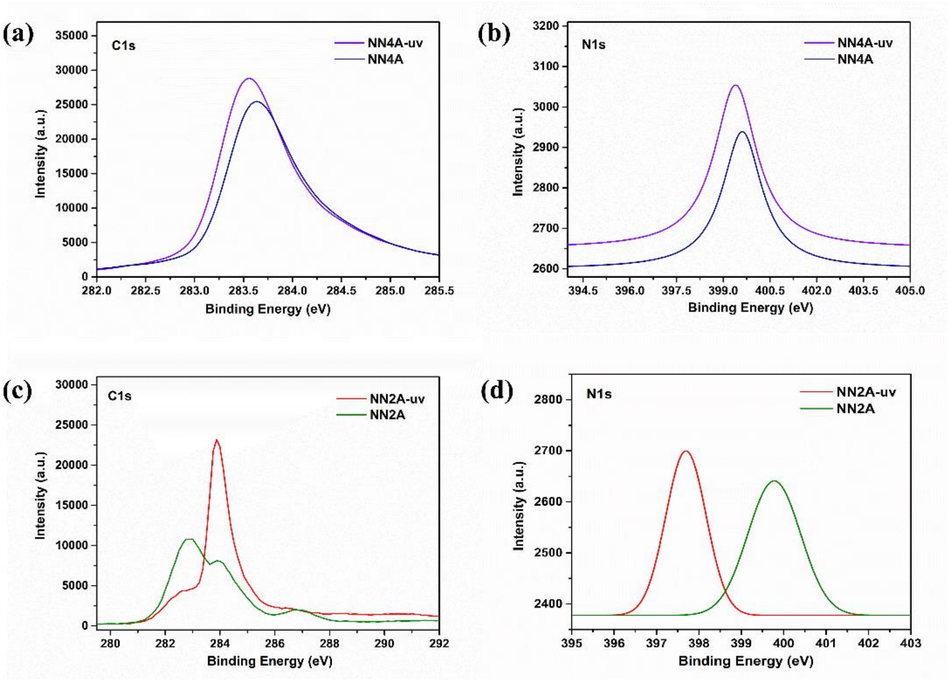

Figure S1. The X-ray photoelectron spectroscopy of self-assembled systems under light-regulated: (a) The C1s spectra of NN4A&NN4A-uv; (b) The N1s spectra of NN4A&NN4A-uv; (c) The C1s spectra of NN2A&NN2A-uv; (d) The N1s spectra of NN2A&NN2A-uv.

In addition, we also experimentally tested the infrared absorption spectrum, and the results are listed in **Figure S2**. We found that the peak at 1700cm^{-1} and the peak at 1200cm^{-1} of NN4A molecular membrane were enhanced after receiving light stimulation. However, the peak at 1700cm^{-1} of NN2A was weakened after receiving light irradiation, the peak decreases to almost disappear at 3000cm^{-1}.

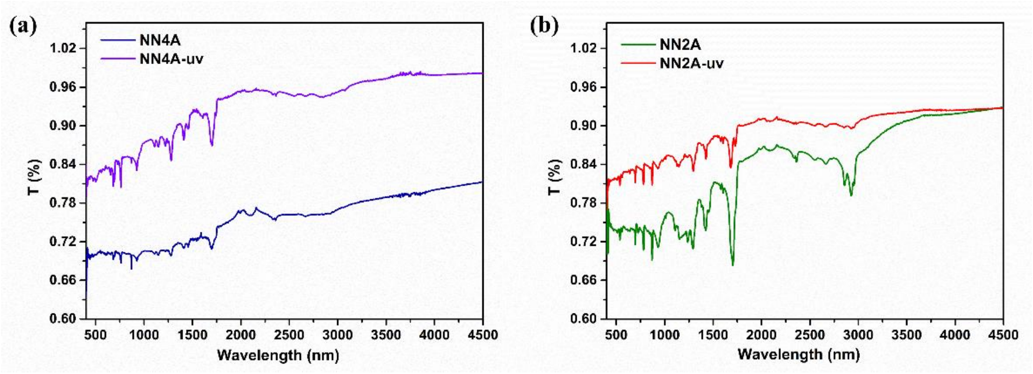

Figure S2. ATR-FTIR spectra of self-assembled systems under light-regulated: (a) NN4A&NN4A-uv; (b) NN2A&NN2A-uv.

Then we also tested and explored the contact angle on the self-assembled molecular layer surface of the two samples, which are shown in **Figure S3**. The results showed that the contact angle of the surface of NN4A molecular layer decreased from 93.6˚ to 82.2˚ under light stimulation, and that of NN2A molecular layer changed from 103˚ to 96.7˚ after light stimulation.

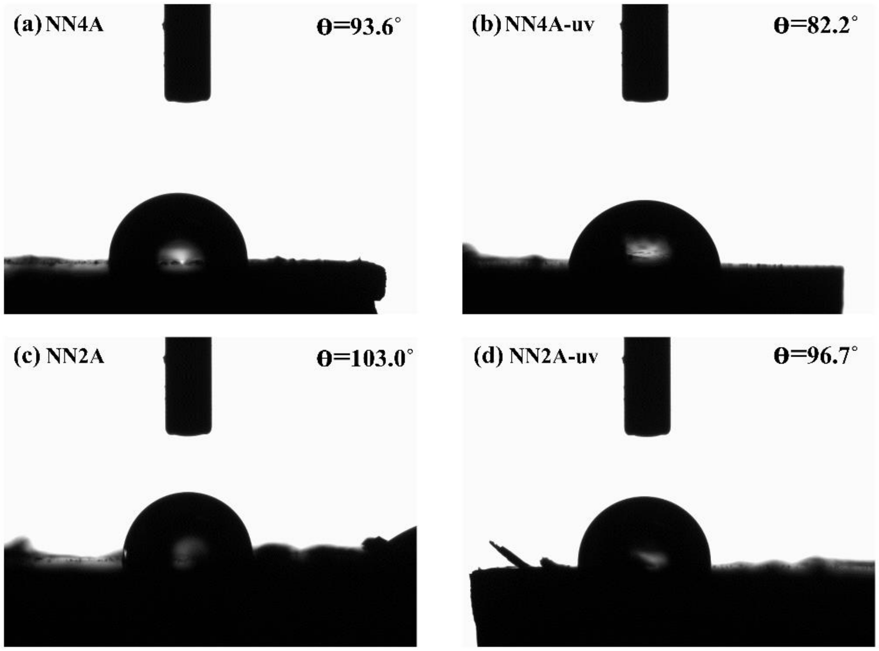

Figure S3. Contact angle of self-assembled systems under light-regulated: (a) NN4A; (b) NN4A-uv; (c) NN2A; (d) NN2A-uv.

Finally, we also used spectroscopic ellipsometry to explore the optical parameters of the two molecular film surfaces before and after light control. The results are shown in **Figure S4**. It was found that the changes of PSI and Delta on the membrane surface of NN4A molecular film were relatively small before and after light stimulation, while the changes of NN2A molecular film were larger after light stimulation. The changes of PSI and Delta of both NN4A and NN2A were all showed a decreasing trend after exposure.

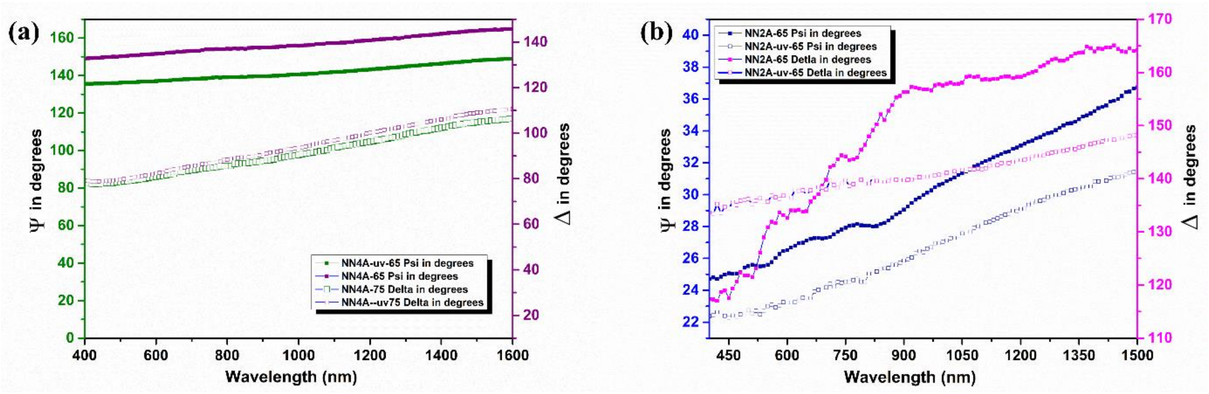

Figure S4. PSI and Delta of self-assembled systems under light-regulated: (a) NN4A&NN4A-uv; (b) NN2A&NN2A-uv.

In summary, the various characterization methods all verified that the self-assembled molecular films changed after being stimulated by light. It is shown that light stimulation has distinguished effect on surface regulation.

2 Characterization of carboxylic azobenzene materials in bulk phase

The photo-stimulatory responses of two selected carboxylic azobenzene compounds in the volume phase were characterized using a dynamic light scattering apparatus. The experimental results are shown in **Figure S5**. The selected solvent is chromatographic pure acetonitrile. It can be seen that the particle size of NN4a substance in the solution changed from 38nm to 18nm after being stimulated by light, and the particle size of NN2A substance in the solution changed from 106nm to 60nm after being stimulated by light. The reason of that is the light isomerized azobenzene molecule, the length of the molecule became shorter. It is proved that the carboxylic azobenzene molecule has obvious isomerism under light stimulation.

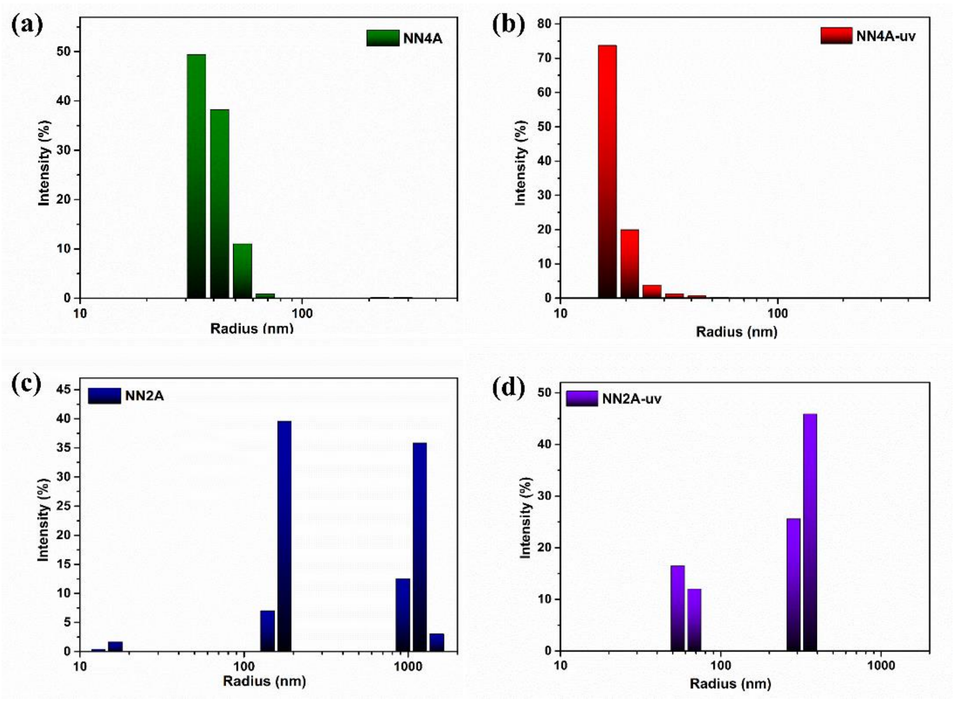

Figure S5. Size distribution of two substances in acetonitrile solution under light-regulated: (a) NN4A; (b) NN4A-uv; (c) NN2A; (d) NN2A-uv.

3 Fatigue resistance of self-assembled molecular layer systems

We have carried out the fatigue resistance experiments on the light-controlled friction performance of the self-assembled molecular membrane systems, and the results are shown in the text. We also carried out experiments on the frictional properties of the molecular film to verify the reversibility of the molecular layer system under repeated displacement irradiation of ultraviolet (UV) and visible light (Vis). The results are listed in **Figure S6**. In summary, it can be seen that the molecular membrane systems of the two substances have distinguished fatigue resistance.

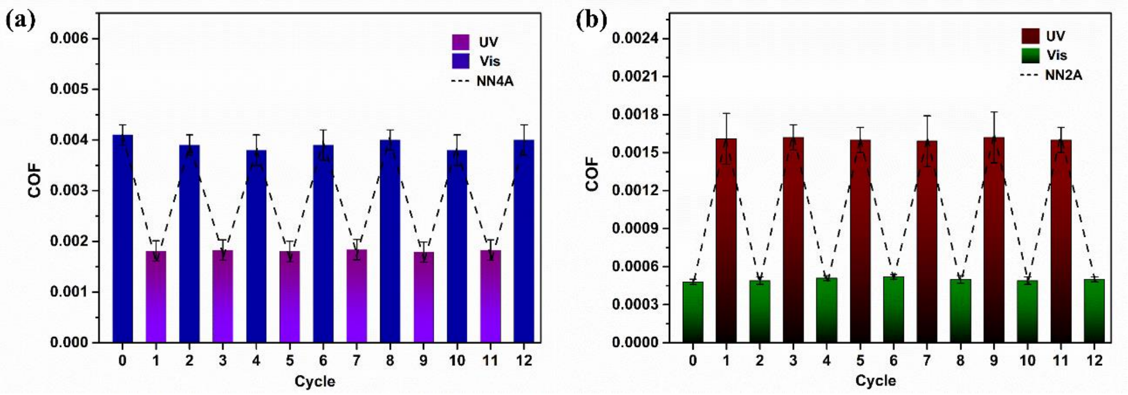

Figure S6. Fatigue resistance of self-assembled systems under light-regulated: (a) NN4A&NN4A-uv; (b) NN2A&NN2A-uv.
